# Effect of Insecticide Resistance on Development, Longevity and Reproduction of Field or Laboratory Selected *Aedes aegypti* Populations

**DOI:** 10.1371/journal.pone.0031889

**Published:** 2012-03-14

**Authors:** Ademir Jesus Martins, Camila Dutra e Mello Ribeiro, Diogo Fernandes Bellinato, Alexandre Afranio Peixoto, Denise Valle, José Bento Pereira Lima

**Affiliations:** 1 Laboratório de Fisiologia e Controle de Artrópodes Vetores, Instituto Oswaldo Cruz, Fiocruz, Rio de Janeiro, Rio de Janeiro, Brazil; 2 Laboratório de Entomologia, Instituto de Biologia do Exército, Rio de Janeiro, Rio de Janeiro, Brazil; 3 Pronex - Rede Dengue, CNPq, Rio de Janeiro, Rio de Janeiro, Brazil; 4 Instituto Nacional de Ciência e Tecnologia em Entomologia Molecular (INCT-EM), CNPq, Rio de Janeiro, Rio de Janeiro, Brazil; 5 Laboratório de Biologia Molecular de Insetos, Instituto Oswaldo Cruz, Fiocruz, Rio de Janeiro, Rio de Janeiro, Brazil; Kansas State University, United States of America

## Abstract

*Aedes aegypti* dispersion is the major reason for the increase in dengue transmission in South America. In Brazil, control of this mosquito strongly relies on the use of pyrethroids and organophosphates against adults and larvae, respectively. In consequence, many *Ae. aegypti* field populations are resistant to these compounds. Resistance has a significant adaptive value in the presence of insecticide treatment. However some selected mechanisms can influence important biological processes, leading to a high fitness cost in the absence of insecticide pressure. We investigated the dynamics of insecticide resistance and its potential fitness cost in five field populations and in a lineage selected for deltamethrin resistance in the laboratory, for nine generations. For all populations the life-trait parameters investigated were larval development, sex ratio, adult longevity, relative amount of ingested blood, rate of ovipositing females, size of egglaying and eggs viability. In the five natural populations, the effects on the life-trait parameters were discrete but directly proportional to resistance level. In addition, several viability parameters were strongly affected in the laboratory selected population compared to its unselected control. Our results suggest that mechanisms selected for organophosphate and pyrethroid resistance caused the accumulation of alleles with negative effects on different life-traits and corroborate the hypothesis that insecticide resistance is associated with a high fitness cost.

## Introduction

Insecticide resistance is one of the major obstacles in the attempt to control disease vectors and agricultural pest insects. The continuous insecticide pressure can select insect populations with 1) an altered integument, less permissive to the insecticide penetration, 2) modifications in the structure or expression level of naturally detoxifying enzymes, 3) mutations in the insecticide target molecules, in the central nervous system, that hamper interaction with the insecticide, or 4) behavioral changes, such as avoiding contact with the insecticide [1]. While some modifications conferring a selective advantage spread quickly in a given population under insecticide pressure, they can be associated with a high fitness cost. In general, genes that rapidly increase in frequency as a consequence of adaptation to a new environment have a fitness cost in the original population habitat [2], [3], [4]. This is probably the consequence of reallocation of resources, affecting physiologic and reproductive processes [5].

In the case of insect vectors, insecticide resistance can interfere with their vectorial capacity, a condition estimated through biological (development and reproduction), ecological and behavioral parameters [6]. Controlled insecticide selection in the laboratory enables the study of resistance dynamics as well as its effects over several adaptive parameters. This was the case for *Aedes aegypti* specimens resistant to the pyrethroid permethrin [7] or submitted to DDT selection, the last resulting in the decrease of both egg production and larvae hatching and survival rates [8].

Brazil presents a growing number of dengue cases, mainly due to the low efficacy of actions to control its vector, based on the use of organophosphates and pyrethroids, respectively against larvae and adults [9], [10], [11]. Reports of *Ae. aegypti* pyrethroid resistance in the country point to both metabolic and target site resistance mechanisms [11], [12]. We investigated, for five Brazilian *Ae. aegypti* populations exhibiting different levels of resistance to pyrethroids and organophosphates, a series of life-trait parameters related to their vectorial capacity, such as larval developmental time, sex ratio, adult male and female longevity, relative amount of blood ingested by females, number of egglaying females, number and viability of eggs. Additionally, we selected one population during nine generations with deltamethrin, the pyrethroid presently employed in the country for the control of adults. During this process, the same aforementioned parameters were followed. We showed that a series of deleterious effects were selected together with resistance in laboratory conditions, while only a discrete commitment was noted in field populations. The implications of these results to vector control are discussed.

## Results

### 1. Laboratory pyrethroid selection and resistance evaluation

**Figure 1:**
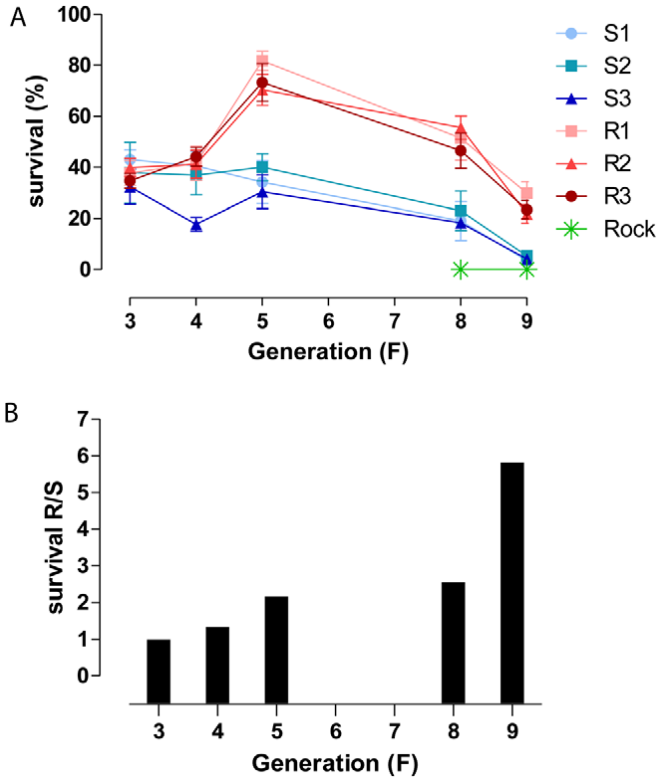
Pyrethroid selection process in the laboratory. Survival dynamics of an *Aedes aegypti* field population submitted to controlled selection in the laboratory. **A** – Survival rate 24 h after exposure to deltamethrin during 1 h (1.5 µg/bottle at F3 and 3.0 µg/bottle afterwards). In R lineages (R1, R2 and R3), adult females were selected with deltamethrin at each generation; S lineages (S1, S2 and S3) were reared in parallel, without insecticide selection. Rockefeller strain was exposed to the same bioassay at F_8_ and F_9_ generations. Mean and standard error are represented. **B** – Survival ratio of the pool of R lineages as compared to their S counterparts at each generation; comparison of mean values (A).

*Aedes aegypti* adult females from Natal/RN were selected with 1.5 µg deltamethrin / bottle from F_1_ (48% mortality) to F_3_ (20–34% mortality). Deltamethrin dosage was then increased to 3.0 µg/bottle. The independent S (S1, S2 and S3) and R (R1, R2 and R3) lineages followed the same tendency within each group (Figure 1-A). S females tended to become progressively more susceptible to deltamethrin. By contrast, survival of R lineages tended to increase at least up to the 5^th^ generation. Surprisingly, this trend was then reversed. From F_5_ on, the survival rate was significantly different between S and R groups (Table S1). It is noteworthy that the survival ratio of R group over S group increased throughout generations (Figure 1–B, Table S1).

### 2. Fitness cost: development and longevity of laboratory selected groups

#### Dynamics of larval development

**Figure 2:**
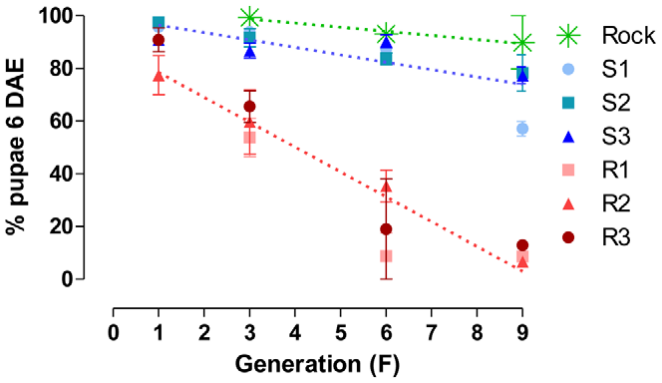
Time of larval development – selected lineages. Mean rate of pupa formation of an *Aedes aegypti* field population submitted to pyrethroid selection in the laboratory. Standard error is also indicated. Lines are regression at the 6^th^ day after larva hatching for pooled lineages of R (dotted red), S (dotted blue) and Rockefeller (dotted green). Values for linear regression analysis are: Rock (β = −1.565, r2 = 0.285, p>0.05), S (β = −2.817, r2 = 0.6095, p<0.0001) and R (β = −9.434, r2 = 0.8217, p<0.0001).

Time needed to complete larval development was progressively increased over generations in the R group. Figure 2 presents the rate of pupae formation at the 6^th^ day after egg hatching (DAE) throughout generations. At the end of the selection process (F_9_), a total of respectively 79 and 11% of pupae were formed in S and R groups. There is a significant progressive delay in larval development of R lineages (up to two days until pupa formation, see Figure S1).

#### Sex ratio

The observed sex ratio was in accordance with the 1∶1 expected values in all groups and generations evaluated (Table S2).

#### Adult longevity

In all cases, including Rockefeller strain, females lived longer than males. A tendency of longevity reduction in the R females was observed (Figure S2). Comparisons among Rockefeller, S and R groups were performed at 30 and 60 days after adult emergence (Table 1). On the 30^th^ day, groups were equivalent up to F_6_. Difference between S and R was significant only for F_9_ females. By contrast, on the 60^th^ day, the higher survival rate of Rock compared to S and R was evident in all generations. In this case a lower survival rate of R females, compared to S ones, was observed in F_6_ and F_9_ (Table 1).

**Table 1:** Adult longevity of an *Aedes aegypti* field population submitted to controlled pyrethroid selection in the laboratory.

	generation	days after adult emergency					
		30^th^ day			60^th^ day		
		Rock	S	R	Rock	S	R
males	F_3_	60.4±3.1^a^	58.5±2.1^a^	58.5±4.1^a^	15.6±3.6^a^	5.71±1.3^b^	2.6±1.2^b^
	F_6_	64.0±2.6^a^	58.6±1.6^a^	57.2±2.6^a^	22.4±3.9^a^	12.3±1.0^b^	8.7±1.4^b^
	F_9_	64.3±10.3^a^	61.9±2.5^a^	51.7±2.5^a^	28.4±8.1^a^	9.8±3.2^b^	8.7±2.2^b^
females	F_3_	82.5±1.3^a^	85.7±2.5^a^	84.1±1.5^a^	30.6±5.6^a^	15.7±1.8^b^	6.5±3.0^b^
	F_6_	81.4±3.0^a^	74.4±2.1^a^	65.4±5.2^a^	35.7±1.8^a^	21.6±2.1^b^	8.6±1.7^c^
	F_9_	81.6±2.2^a^	81.2±2.1^a^	61.9±1.6^b^	40.0±1.2^a^	22.9±2.7^b^	8.9±1.1^c^

Values represent percent survival rate±standard error, 30 or 60 days after adult emergence. Rock, R and S refer, respectively, to Rockefeller strain and groups derived from Natal population selected or not with deltamethrin. For each generation and for each day evaluated, values followed by the same letter do not differ significantly (p>0.05, ANOVA followed by Tukey's Multiple Comparison Test).

#### Blood feeding

The proportion of ingested blood relative to the body weight varied significantly between S and R groups since F_3_ (Table 2 and Figure S3).

**Table 2:** Increase of *Aedes aegypti* adult female weight after a blood meal; comparison among R, S and Rockefeller.

	Rock		S		R	
Generation	n	weight ratio	n	weight ratio	n	weight ratio
F_1_	3	1.9±0.18 ^a^	9	1.7±0.12 ^a^	6	1.6±0.09 ^a^
F_3_	6	1.7±0.04 ^a^	18	1.4±0.04 ^b^	18	1.3±0.03 ^c^
F_6_	6	2.0±0.04 ^a^	17	1.8±0.02 ^a^	18	1.3±0.04 ^b^
F_9_	3	2.2±0.02 ^a^	15	2.0±0.08 ^a^	15	1.4±0.03 ^b^

Ratios were obtained by comparison of females weight after and before the blood meal. Values indicate mean and standard error. n: number of pools of 10 females. Comparisons were performed at each generation; values followed by the same letter do not differ significantly (p>0.05, ANOVA, followed by Bonferroni's Multiple Comparison Test).

### 3. Fitness cost: oviposition and egg viability of laboratory selected groups

**Figure 3:**
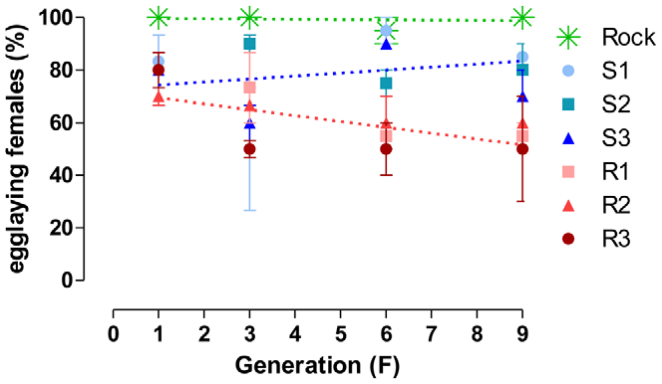
Amount of egglaying females – selected lineages. Mean rate of *Aedes aegypti* egglaying females during deltamethrin selection in the laboratory. All blood-fed females were considered. Standard error is also indicated. Lines indicate linear regression analysis for Rock (β = −0.0996, r2 = 0.0332, p>0.05), and pooled S (β = 1.136, r2 = 0.0691, p>0.05) and R (β = −2.228, r2 = 0.4114, p<0.05) lineages.

**Figure 4:**
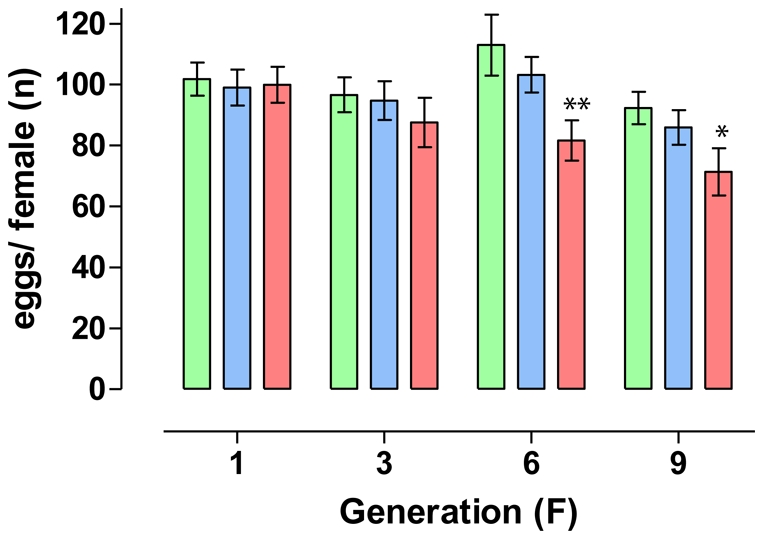
Number of eggs – selected lineages. Number of eggs per egglaying *Aedes aegypti* female during deltamethrin selection in the laboratory. Green, blue and red bars indicate respectively Rock, S and R females. Standard error is also indicated. Values significantly or highly significantly different from Rock are indicated by (*) or (**), respectively.

**Figure 5:**
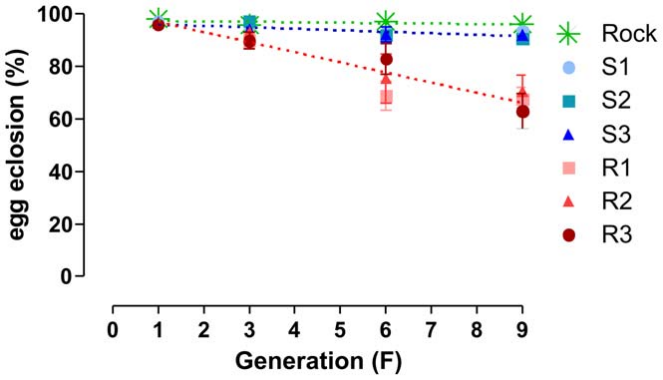
Eggs viability – selected lineages. Egg viability of *Ae. aegypti* during deltamethrin selection in the laboratory. Mean egg hatching rate and standard error are represented. Lines are regression of Rock and the pooled S and R lineages. Values for linear regression analysis are: Rock (β = −0.1644, r2 = 0.2748, p>0.05), S (β = −0.5666, r2 = 0.5212, p<0.05) and R (β = −3.854, r2 = 0.8998, p<0.0001).

The rate of egglaying females was lower in S and R groups since F_1_ generation, when compared to Rock (Figure 3). Moreover, in the R group the proportion of egglaying females decreased along the selection process: at the F_9_ generation, a reduction of roughly 20 and 45% is observed in the groups S and R, respectively, when compared to Rockefeller (Table 3). The number of eggs laid was also significantly affected in the R group, differing from Rock values at F_6_ and F_9_ generations (see Figure 4 and Table S3). In the F_9_ generation, when compared to Rock, a reduction of 22.6% in the number of eggs per female was noted in the R group, contrasting with a 6.8% reduction in the S group (Table 3). Eggs viability profile presented the same tendency (Figure 5), with a significant reduction (roughly 30%) for R group at F_9_ generation (Table 3).

**Table 3:** Life-trait parameters of S and R lineages at F9 generation in comparison with the Rockefeller strain.

	Rock X selected lineage (F_9_)			average reduction (%)		
parameter	Rock	S	R	S/ Rock	R/ Rock	R/ S
larval development rate^1^	89.9±14.3 ^a^	70.9±11.9 ^a^	9.4±2.9 ^b^	21.1	89.5	86.7
♂ longevity rate 30^th^ day^2^	64.3±14.5 ^a^	61.9±6.7 ^a^	51.7±6.1 ^a^	3.7	24.4	16.5
♀ longevity rate 30^th^ day^2^	81.6±3.0 ^a^	81.2±5.2 ^a^	61.9±3.9 ^b^	0.5	24.1	23.8
bloodmeal engorgement^3^	2.2±0.1 ^a^	2.0±0.3 ^a^	1.4±0.1 ^b^	10.0	40.0	30.0
rate of egglaying ♀^4^	100.0±0 ^a^	81.7±2.9 ^b^	55.0±5.0 ^c^	18.3	45.0	32.7
eggs/female^5^	92.3±24.0 ^a^	86.0±38.0 ^a^	71.4±46.4 ^b^	6.8	22.6	17.0
eggs viability rate^6^	96.0±1.0 ^a^	92.0±4.7 ^a^	67±17.4 ^b^	4.2	30.2	27.2

Mean ± standard deviation are shown in all cases. Numbers followed by the same letters do not differ significantly (ANOVA, p>0.05). The last two columns (“average reduction”) compare S and R groups with the Rockefeller strain and R with S group.

(1) Larvae development is the rate of pupae formed up to the 6^th^ day after larval hatching.

(2) Longevity is represented by the mean survival rate at the 30^th^ day after adult emergence.

(3) Blood meal engorgement refers to the females' weight after / before bloodfeeding.

(4) Percentage of blood fed females that laid eggs.

(5) Mean number of eggs laid by bloodfed females.

(6) Rate of larvae hatching.

A general decline in the performance of nearly all parameters evaluated was noted for both S and R groups at the end of the selection process (F_9_). In all cases, a decrease was more prominent for R group (Table 3). Larval development was the most affected aspect: on the 6^th^ day after larval hatching only 10% of pupae were observed in the R group, in contrast to 90 and 70% for Rockefeller and S group, respectively.

### 4. Resistance to organophosphate of laboratory selected groups

Temephos dose response bioassays revealed resistance ratios (RR_95_) of 26.0 and 15.6 in respectively R and S groups at the F_9_ generation. In contrast, RR_95_ of the original field population was 18.6 [11], suggesting that pyrethroid laboratory selection resulted in cross resistance with the organophosphate.

### 5. Fitness cost: development and reproduction of field populations

**Figure 6:**
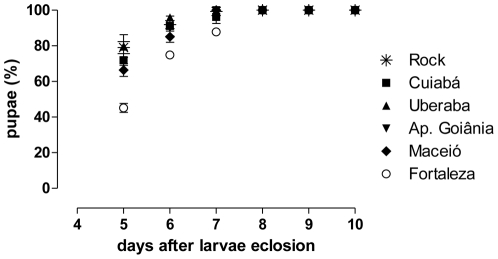
Time of larval development – field populations. Larvae to pupae developmental time of five natural populations. Cumulative mean percentage of pupae formation and standard error.

**Figure 7:**
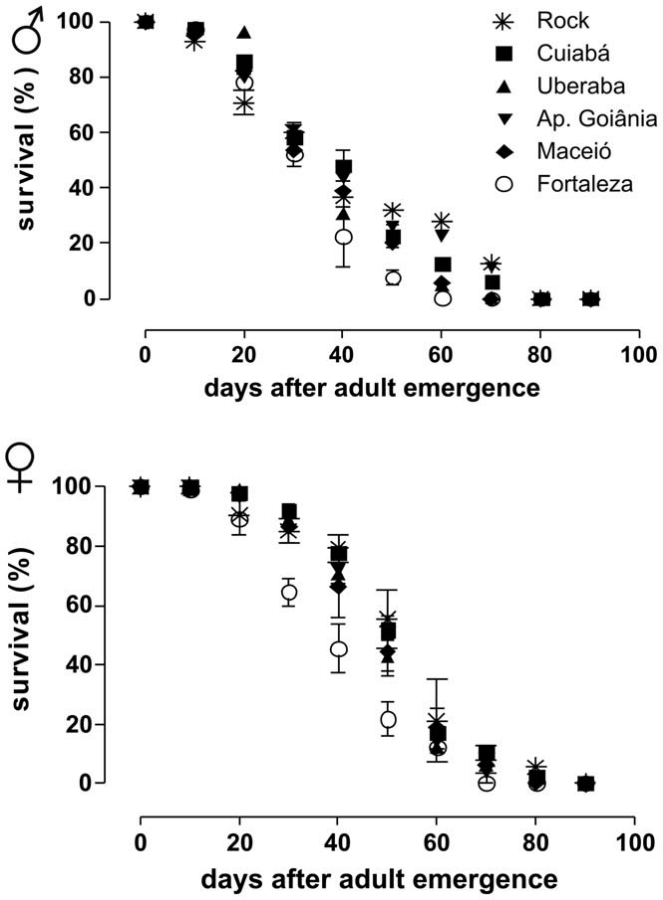
Adult longevity – field populations. Longevity of adults from five natural populations under laboratory conditions. Rockefeller strain (Rock) was used as an internal control of the assay. Mean percentage and standard error are presented.

**Figure 8:**
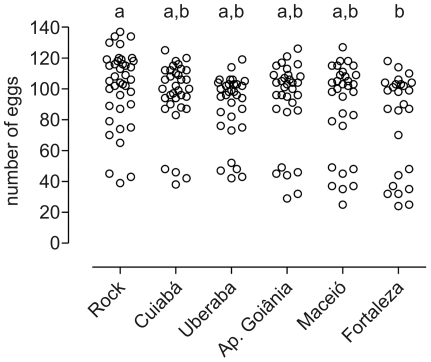
Number of eggs – field populations. Number of eggs per egglaying female of *Aedes aegypti* F1 field population and Rockefeller. Each dot indicates the number of eggs from an individual female. Identical letters above the scatter dots mean non-significant differences according to One Way ANOVA followed by Tukey Multiple Comparison Test.

Among the five field populations evaluated, a delay in larval development was observed only in Fortaleza mosquitoes, whose pupae formation rate differed from the Rock strain at the 5^th^ (q = 7.994; p<0.01) and 6^th^ days (q = 5.777, p<0.05) after larvae hatching (Figure 6). Fortaleza adults also exhibited the lowest longevity (Figure 7). Nevertheless, compared to Rock, this reduction was only evident by the 50^th^ day after adult emergence in the case of males (q = 5.777, p<0.01, Figure 7A). This effect was more pronounced among females: differences were already noticeable on the 30^th^ day (q = 5.726, p<0.05, Figure 7B). Similarly to the laboratory selected lineages, in no case significant differences in the sex ratio were detected (data not shown). The amount of ingested blood did not differ significantly among populations and Rockefeller (F = 1.1779, p = 0.1917), ranging from 1.8 to 2.1 times the female weight. Notwithstanding, although Rockefeller exhibited a slightly higher number of eggs compared to natural populations, significant differences were noted only between Rock and Fortaleza (mean diff. = 32.5, t = 5.3, p<0.05; Figure 8). There was no significant difference in the rate of egg hatching among populations and Rock (data not shown). The egg hatching rate was 81.5 to 94.0% respectively for Fortaleza and Rockefeller, but this difference was not significant between them (ANOVA, followed by Tukey Multiple Comparison Test, p>0.05).

Taken together, our data reveal that among field populations, only Fortaleza mosquitoes exhibited significant reduction of some developmental and reproductive parameters. In contrast, mosquitoes submitted to a controlled pyrethroid selection, under laboratory conditions, presented differences in almost all evaluated aspects (Table 3).

## Discussion

In this study we evaluated several life-trait parameters of six Brazilian field *Ae. aegypti* populations, exhibiting different insecticide resistance status. Five of them were assayed in the F1 generation and one during the process of pyrethroid selection in the laboratory. The latter revealed a series of increasing deleterious effects on both development and reproduction in the course of pyrethroid selection, with consequent reduction of the general fitness. Notwithstanding, we also observed, from the F*_5_* generation on, a reversal of the resistance status, in spite of pyrethroid selection. This is in contrast with other reports, especially when *Ae. aegypti* resistance is measured in the larval stage [7], [13], [14], [15], [16]. It is noteworthy that Kumar et al. [13], working with insecticide selection for 40 generations, only detected high resistance levels when *Ae. aegypti* larvae, but not adults, were employed. In contrast, the selection process described here was based on deltamethrin exposure of adult females exclusively. Therefore, it is possible that less resistant males with putative higher mating success have contributed to the maintenance of susceptible alleles during the selection process, decreasing its efficiency. Nevertheless, the decrease in general fitness observed in the selected strains suggests accumulation of deleterious effects due to pleiotropy of the resistant alleles or to a hitch-hiking effect [17]. In this latter case, it may occur when a resistance gene positively selected is on linkage disequilibrium with other loci, which when in homozygosis can present a fitness cost.

In spite of the absence of a clear gain in the pyrethroid resistance status in the deltamethrin selected lineage, increase in resistance to the organophosphate temephos was evident. Sampling, which originated the S and R groups here presented was performed on 2005 in the municipality of Natal/RN. Resistance monitoring in the previous year revealed a RR_95_ of 18.6 for temephos in this locality [11]. Controlled pyrethroid selection for nine generations, with group R, increased the RR_95_ up to 26.0 for the organophosphate temephos. On the other hand, group S, reared in parallel but without insecticide selection, had its RR_95_ diminished to 15.6. In the field, temephos is no longer used in Natal /RN since 2005, when it was replaced by *Bti*. According, data from 2007 collections showed a decreased RR_95_, of 10.4 [18].

Taken together, our data strongly suggest that pyrethroid selection induced cross resistance to the organophosphate temephos, presumably as a function of increase in the activity of metabolic resistance mechanisms, based on detoxifying enzymes. Accordingly, several natural Brazilian *Ae. aegypti* populations, exposed to organophosphates for three decades, developed cross resistance to pyrethroids, probably due to the increase in the activity of Esterases and Glutathione-S-transferases [11]. In Natal, the field population used for laboratory selection, the target site kdr mutation Val1016Ile was not detected [19]. Although in principle this corroborates the putative involvement of detoxifying enzymes in insecticide resistance, we are also presently looking for other alterations on the pyrethroid target site, potentially involved with both resistance and a high fitness cost. A recent temephos selection of an *Ae. aegypti* lineage, under laboratory conditions during 17 generations, resulted in a 25 fold increase of the resistance ratio to this insecticide. Increased activity of detoxifying enzymes was primarily selected; no target site related alterations were noticed [20]. The authors also showed that resistance can be highly diminished or even extinct after only few generations without selection pressure. It seems that when the selected mechanisms are related to metabolic resistance, the susceptible status is prone to be recovered when insecticide pressure ceases. These results agree with the assumption that most of the mechanisms selected for insecticide resistance are generally associated to fitness disadvantages. In a very recent report, six *Ae. aegypti* field populations were selected with pyrethroid for five generations in the lab [21]. A progressive increase in resistance levels was observed (mainly based on kdr mutation), except for two populations, in which resistance dropped in the fourth generation. Besides, a third population was lost due to low oviposition rates in the second generation of pyrethroid selection. In both situations, the authors attributed these events either to accumulation of deleterious effects or to the presence of recessive alleles linked to kdr mutation, or even to metabolic resistance related genes.

Investigations regarding fitness cost, or life history, are generally performed through comparisons of biological parameters, such as developmental kinetics, fecundity, or even growing rates, under controlled laboratory conditions [22]. In the present work, the Rockefeller strain, kept for decades under laboratory conditions, was employed as an internal control of all the assays. Besides comparisons between pyrethroid selected and non selected groups for nine generations, we also investigated several life-trait parameters of five *Ae. aegypti* F1 natural populations. One of these populations, a district of Fortaleza municipality, had an unusual high temephos resistance ratio, certainly derived from the field collection strategy employed. In this case it was limited to a neighborhood highly infested, and in consequence strongly pressured with insecticide. A strengthened selection of resistant specimens was then expected. Accordingly, among the field populations here evaluated, only Fortaleza mosquitoes exhibited significant commitment of a few life history traits. In this regard, it should also be mentioned that the Brazilian Northeast Region in general, and the municipality of Fortaleza in a more particular context, have a historic of intense use of insecticides.

Factors selected for resistance may present direct pleiotropic effects in one or few life-trait aspects. In fact alterations in different traits can be considered manifestations of the insect physiological commitment to face the challenge represented by insecticide exposure. For instance, time of development is a primary aspect of fitness in disseminating mosquito populations [23]. In the presence of natural predators or parasites, every delay in development has the potential to reduce the survival rate of larval instars [24]. Here we show a progressive delay in the larval development of the pyrethroid selected group (R) from F_1_ to F_9_. By contrast, among the natural populations evaluated, only Fortaleza, the most resistant to the organophosphate temephos (RR_95_ = 43) presented commitment in pupation. Delay in development dynamics was also observed in the Lepidoptera *Spodoptera exigua* selected with the pyrethroid fenvalerate [25] and in two *Cydia pomonella* (Lepidoptera: Tortricidae) strains, selected in the laboratory with the pyrethroid deltamethrin or with the Insect Growth Regulator diflubenzuron [26].

Longevity reduction, mainly in females, was observed in the pyrethroid exposed (R) group along the selection procedure. This is a relevant issue, since adult female longevity, and its duration related to the extrinsic incubation period of a pathogen, is one major aspect of mosquito vectorial capacity. By contrast, shortening in the longevity seemed not to be generally associated with insecticide resistance in field populations. Accordingly, no differences in longevity were observed in some strains of the malaria vectors *Anopheles gambiae* and *Anopheles stephensi* resistant to γ-HCH and dieldrin [27].

The blood meal size is certainly one key parameter regarding both vectorial capacity and general fitness, since since it influences at least two aspects in a directly proportional way: the load of parasites ingested and the number of eggs laid. A significant reduction of the relative amount of ingested blood was observed in the pyrethroid selected R lineages at the F_9_ generation, compared to both Rockefeller and S group. Interestingly, Corbel et al. [28] reported that the amount of blood ingested by the main malaria vector, *An. gambiae*, was inversely proportional to the permethrin dosage in treated nets.

Reduction in the number of egglaying females was also noted for R group, suggesting either a lower fecundity rate or, alternatively, decreased egglaying ability. Mebrahtu et al. [7] observed that the rate of inseminated *Ae. aegypti* females from a field population resistant to permethrin was lower when compared to a susceptible field strain. Additionally, resistant females took longer to lay their eggs. Lower insemination rates observed for γ-HCH and dieldrin resistant *An. gambiae* and *An. stephensi* females were attributed to a lower activity of resistant males [29].

Reduction of both number and viability of eggs laid by pyrethroid selected females (group R) was noted. In contrast to the laboratory selection, evaluation of natural F1 populations revealed that only females from Fortaleza, which were the most resistant to temephos, exhibited reduction in the number, but not in the viability of deposited eggs. There is a series of reports describing reduction in the number of eggs laid by insecticide resistant strains derived from controlled selection experiments. This is the case of fenvalerate resistant *Spodoptera exigua* [25], deltamethrin and diflubenzuron resistant *Cydia pomonella* [26] and DDT resistant *Ae. aegypti* [8]. Mebrahtu et al. [7] also detected reduced larval hatching rate of *Ae. aegypti* derived from permethrin resistant specimens.

The accumulation of negative effects in the life-trait parameters here investigated (due to pleitropic effects of resistance genes or hitch-hiked deleterious alleles) was evident in laboratory selected lineages but rare in natural populations. The deleterious pleitropic effects of resistance alleles might be reduced over time thanks to the selection of modifiers in natural populations subjected to pressure for insecticide resistance,. In addition, the linkage disequilibrium between hitch-hiked deleterious alleles and resistance genes will also tend to decay over time due to recombination. Neither case is very likely to have occurred in the selected lines in only 9 generations. Here we presented a study showing problems related to insecticide resistance in life-trait parameters in a laboratory selected lineage as well as in the F1 of natural populations. Since there are very few options of compounds available for using in the control of insect disease vectors, the knowledge of the biochemical/molecular nature of the deleterious alleles associated to insecticide resistance can be an important tool to investigate the life time of an insecticide.

## Materials and Methods

### 1. Mosquito populations

**Figure 9:**
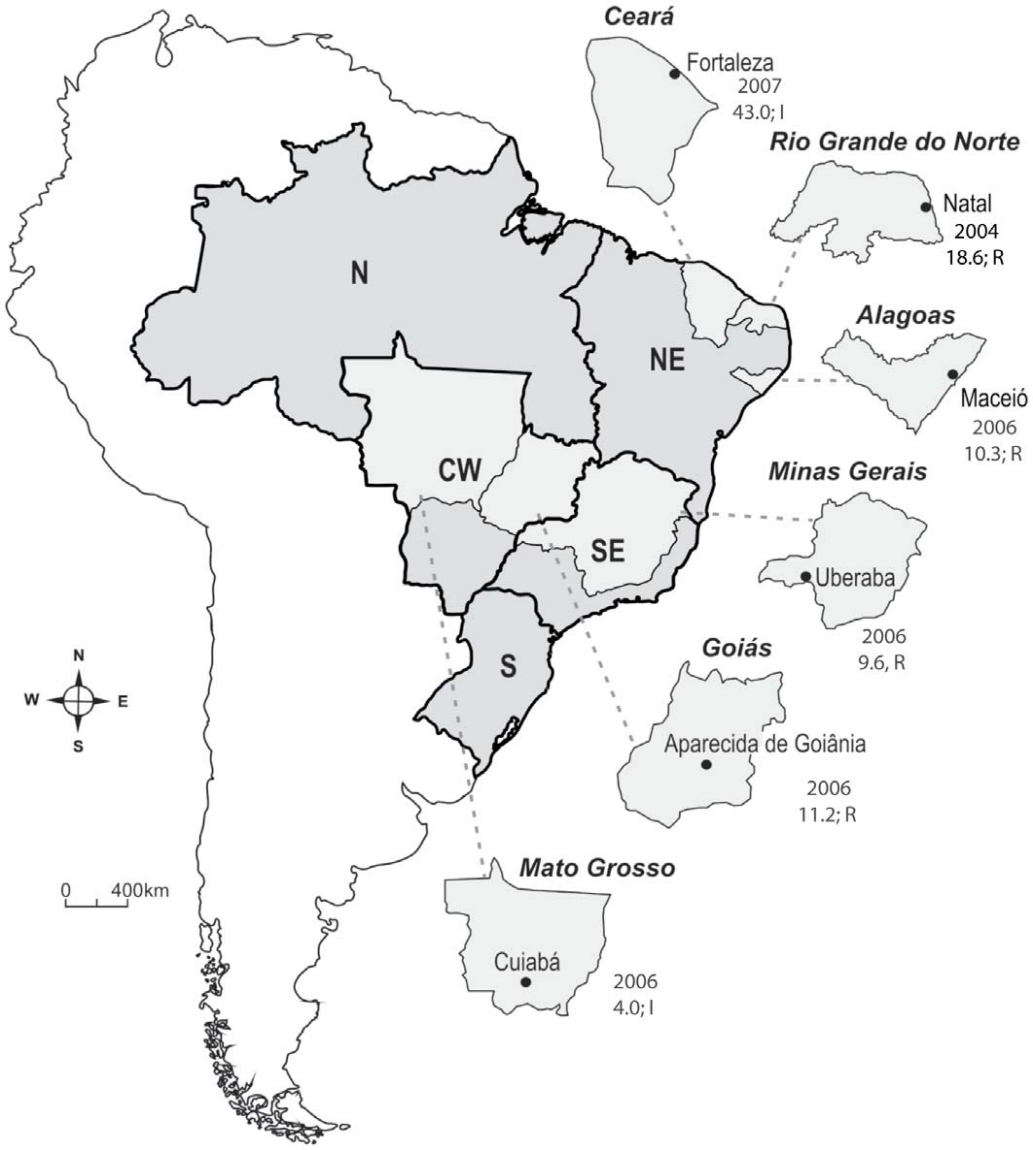
Original localities of *Aedes aegypti* studied populations. Brazilian map showing the States and municipalities used in the study. Bold lines indicate the different geographical regions of the country: N = North, NE = Northeast, SE = Southeast, S = South, CW = Central-Western regions. Numbers below each locality indicate, respectively, the collection year and the Resistance Ratio (RR_95_) to temephos; “R” and “I” refer to the pyrethroid status. See Materials and Methods for details.

The Rockefeller (Rock) strain was established in 1959 in the Rockefeller Institute (New York, NY) [30]. It was used in all assays, and along all generations, as an internal standard control of experimental conditions. Any deviation of Rock results from the expected values would invalidate a given assay. Rock was also employed as an insecticide susceptibility reference strain. Five *Ae. aegypti* field populations, belonging to three Brazilian regions, with different status of susceptibility to organophosphates and pyrethroids, were used at F1 generation: Henrique Jorge-Fortaleza/CE, Maceió/AL, Uberaba/MG, Aparecida de Goiânia/GO and Cuiabá/MT. Controlled pyrethroid selection, in the laboratory, was performed with mosquitoes collected at Natal/RN, Northeast Brazil, in 2005. With exception of Henrique Jorge, a district of Fortaleza, chosen because of its high infestation rates, samples were obtained through collection of eggs with ovitraps installed to cover the whole municipalities, as described elsewhere [10]. In the case of Fortaleza, 40 ovitraps were installed at Henrique Jorge [31]. Details concerning samples location, collection year and insecticide resistance status are presented in Figure 9.

### 2. Mosquitoes rearing

After allowing *Ae. aegypti* eggs to hatch during 24 hours, 500 larvae were transferred to plastic basins (27×19×7 cm) filled with 1 L of dechlorinated water. Larvae were fed with 0.5 g of cat food (Friskies, Purina/ Camaquã/RS), added each third day. Larvae were kept in BOD incubators at 28±1°C. Under these conditions all larvae develop into pupae in 5–6 days. Pupae were transferred to carton cages (17 cm diameter×18 cm high) and resulting adults were fed *ad libitum* with 10% (w/v) sugar solution. Adults were kept under 25±2°C and 70±10% rh. Adult females were fed on anesthetized guinea pigs. Three days after the blood meal oviposition cups, covered internally with a filter paper and partially filled with water, were introduced in the cages to receive oviposition. Two days later oviposition cups were removed from the cages and eggs were stored for further use up to six months after drying.

### 3. Pyrethroid selection

Pyrethroid selection was performed exclusively with female adults, 3–5 days old, exposed to deltamethrin in impregnated glass bottles [9]. The amount of insecticide was frequently calibrated to produce mortality rates around 50%. Selection consisted of 1 hour deltamethrin exposure, followed by a 24-hour recovery period. Surviving (*resistant*) females (R group) were then transferred to carton cages, in three distinct lineages, named R1–R3. Control lineages (S1–S3) consisted of mosquitoes reared in the same conditions, except for the absence of insecticide (S group). Each lineage started with 200 females at every new generation and remained isolated from the others during the whole procedure. Selection was performed during nine uninterrupted generations. In all lineages and for each generation females were allowed to have one blood meal/ week during three weeks.

### 4. Insecticide resistance bioassay

Adult resistance status was evaluated through qualitative bottle bioassays [9]. Each bioassay was repeated with 20–25 females at least three times in different days. Field populations, were exposed to bottles impregnated with 5 ug the pyrethroid deltamethrin (Bayer CropScience Ltda, lot DGDLTK057) dissolved in acetone. Under these conditions, mortality lower than 80% are indicative of resistance (“R” in Figure 9) and values between 80 and 98% reveal an incipient alteration (“I”).

Monitoring of pyrethroid selected groups was performed with bottles impregnated with 1.5 ug deltamethrin at F3 generation and with 3.0 ug afterwards. At least three deltamethrin impregnated bottles were used for each assay and for each lineage.

Dose-response bioassays with the organophosphate temephos were performed according to Lima et al. [10] with L3 larvae from field populations and from the F_9_ generation of the selected lineages. Briefly, larvae were exposed to a series of 7–9 insecticide concentrations, with four replicas per concentration and 20 larvae per replica. Bioassays for each lineage were performed four times in different days. Resistance ratios (RR_95_) were calculated by comparison with Rockefeller values, using probit analysis (software Polo-PC, LeOra Software, Berkeley, CA).

### 5. Fitness: development and longevity evaluation assays

The following assays were performed at least twice; Rockefeller mosquitoes, taken directly from the laboratory colony, were always assayed in parallel, as an internal control.

#### Kinetics of larval development

Eggs were immersed in water during 24 hours to induce hatching. Duplicate samples of 200 larvae each were carefully transferred to plastic basins containing 1 liter of dechlorinated water and reared as detailed in item 4.2. Pupae were daily scored and transferred to cages. The cumulative rate of pupae formed up to the 6^th^ day after larva hatching was evaluated.

#### Sex ratio

Numbers of females and males resulting from the assay described above were compared.

#### Adult longevity

Adult longevity was monitored in duplicate samples of 100 couples each, which were kept in carton cages, fed with sugar solution *ad libitum*. Statistical comparisons of female or male survivorship were performed arbitrarily on the 30^th^ and 60^th^ days after adult emergence.

#### Blood meal size ratio

Five-day old adult females were weighed in an analytical balance (APX – 200, Denver Instrument) before or after feeding in anesthetized guinea pigs during 30 minutes. In each case three pools of 10 females were used. Results are expressed as the ratio between the weights on both conditions.

### 6. Fitness: evaluation of reproductive aspects

Several parameters were considered: a) the ratio of egglaying females; b) the number of eggs per female and c) the egg hatching rate. Oviposition of adult females, three days after a blood meal, was individually stimulated, in 6 cm diameter Petri dishes, covered with a wet filter paper, as described elsewhere [31]. The number of egglaying females was registered 24 hours later. Seven days after the end of oviposition, 10 Petri dishes were randomly chosen. Hatching was stimulated by immersion of the filter papers in organic matter rich water [32], during 24 hours. Only plates exhibiting some larvae eclosion were considered for egg hatching analysis.

### 7. Statistical analysis

Lineages within each laboratory reared group S (S1–3) and R (R1–3) were compared by ANOVA, followed by Tukey's multiple comparison test. When there were no significant differences among lineages of each group, data from groups S or R were pooled. Comparisons among Rockefeller, R and S groups were also made through ANOVA, followed by Tukey's multiple comparison test, except for egg numbers, performed with the non-parametric Kruskal-Wallis test. Numbers of female and male adults were compared by Fisher's exact test.

Except were indicated, similar tests were performed to compare the same parameters among field populations. Analyses were conducted with GraphPad Prism version 5.00 for Windows (GraphPad Software, San Diego California USA).

## Supporting Information

Figure S1 **dynamics of** ***Ae. aegypti*** **pupae formation in R and S lineages and Rockefeller strain.** Mean rate with standard error are shown.
(TIF)

Figure S2 **Longevity of** ***Ae. aegypti*** **adults derived from selection with (R) or without (S) pyrethroid pressure in the laboratory in the F_3_, F_6_ and F_9_ generations.** Mean rates with standard error of alive females (panels A–C) and males (panels D–F) are represented. Rockefeller strain was reared in parallel as an internal control.
(TIF)

Figure S3 **Blood meal ingestion of** ***Ae. aegypti*** **adults resulted from Rock (green) S (blue) and R (red) groups.** Mean and standard error are shown.
(TIF)

Table S1 **Student** ***t*** **test comparison between** ***Aedes aegypti*** **pooled R and S groups survival after exposure to deltamethrin under laboratory selection.**
(DOCX)

Table S2 ***Aedes aegypti*** **sex ratio of S and R groups under laboratory conditions, throughout generations.**
(DOCX)

Table S3 **Number of eggs per** ***Aedes aegypti*** **S and R females in the course of selection with deltamethrin.**
(DOCX)
