# Supplementary figures and images for: Effect of Insecticide Resistance on Development, Longevity and Reproduction of Field or Laboratory Selected Aedes aegypti Populations
Source: PLoS One. 2012 Mar 14;7(3):e31889. doi: 10.1371/journal.pone.0031889 (PMC3303777; doi:10.1371/journal.pone.0031889)

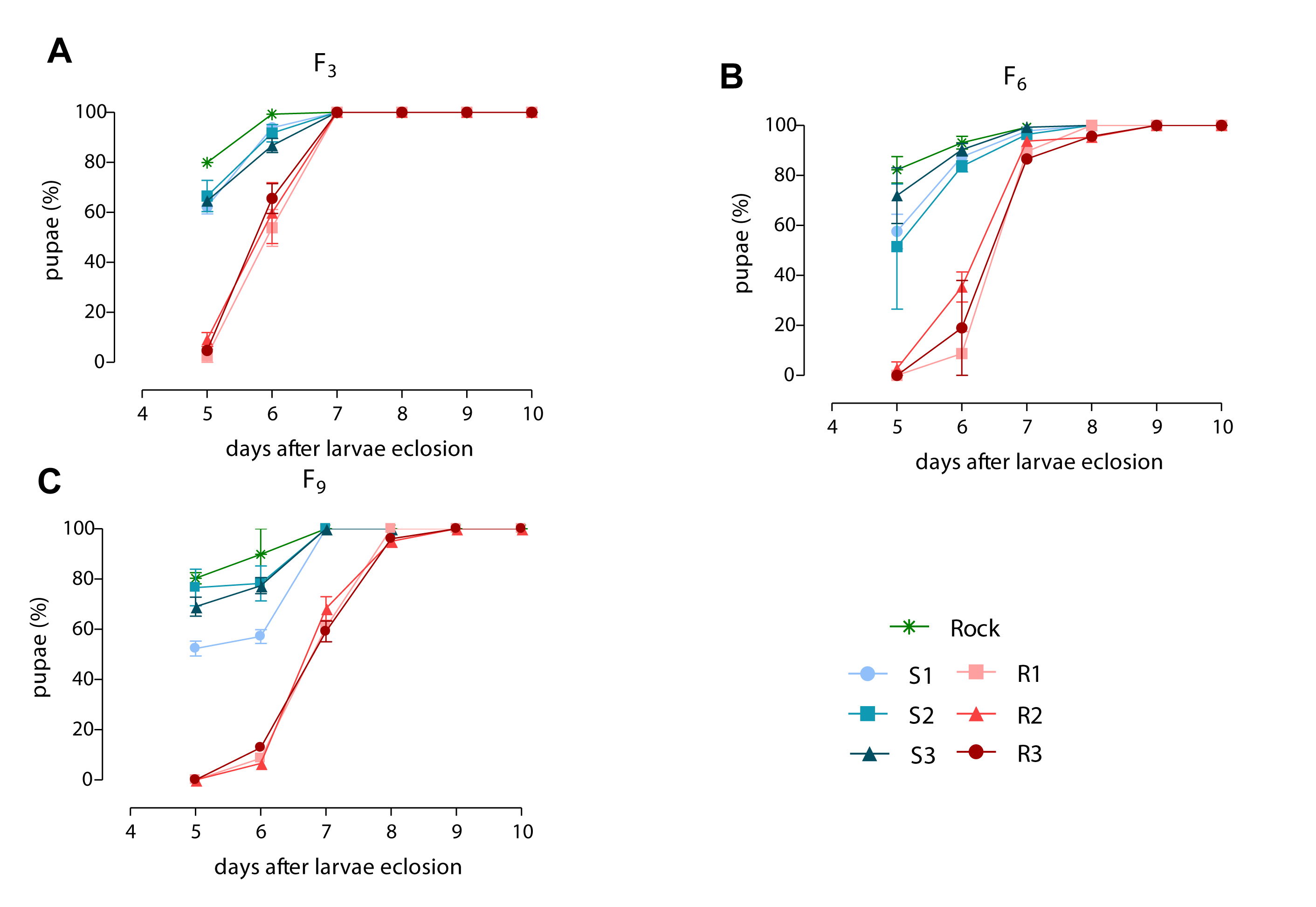

Supplement: Figure S1 — dynamics of Ae. aegypti pupae formation in R and S lineages and Rockefeller strain. Mean rate with standard error are shown. (TIF) [file pone.0031889.s001.tif]

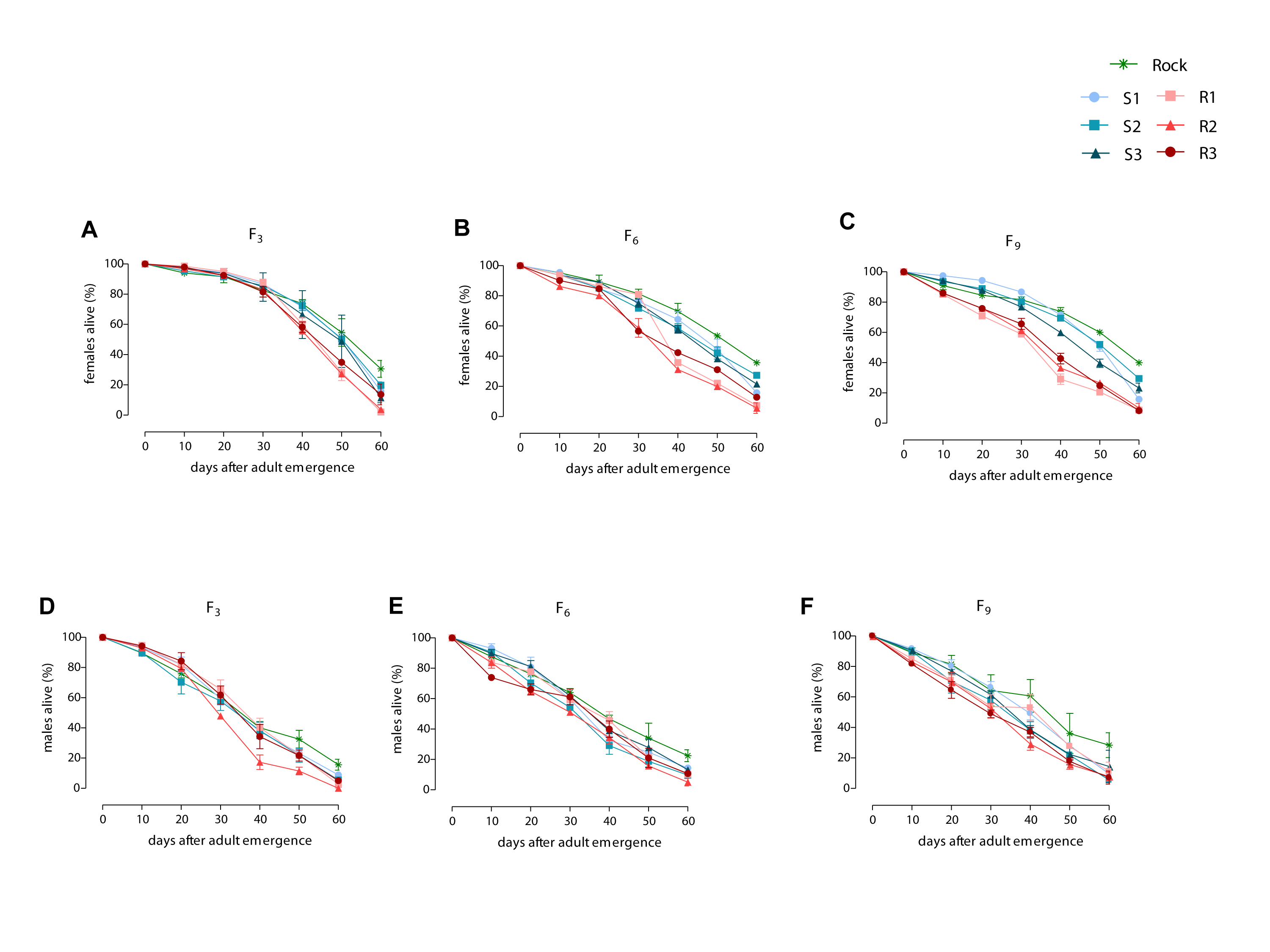

Supplement: Figure S2 — Longevity of Ae. aegypti adults derived from selection with (R) or without (S) pyrethroid pressure in the laboratory in the F3, F6 and F9 generations. Mean rates with standard error of alive females (panels A–C) and males (panels D–F) are represented. Rockefeller strain was reared in parallel as an internal control. (TIF) [file pone.0031889.s002.tif]

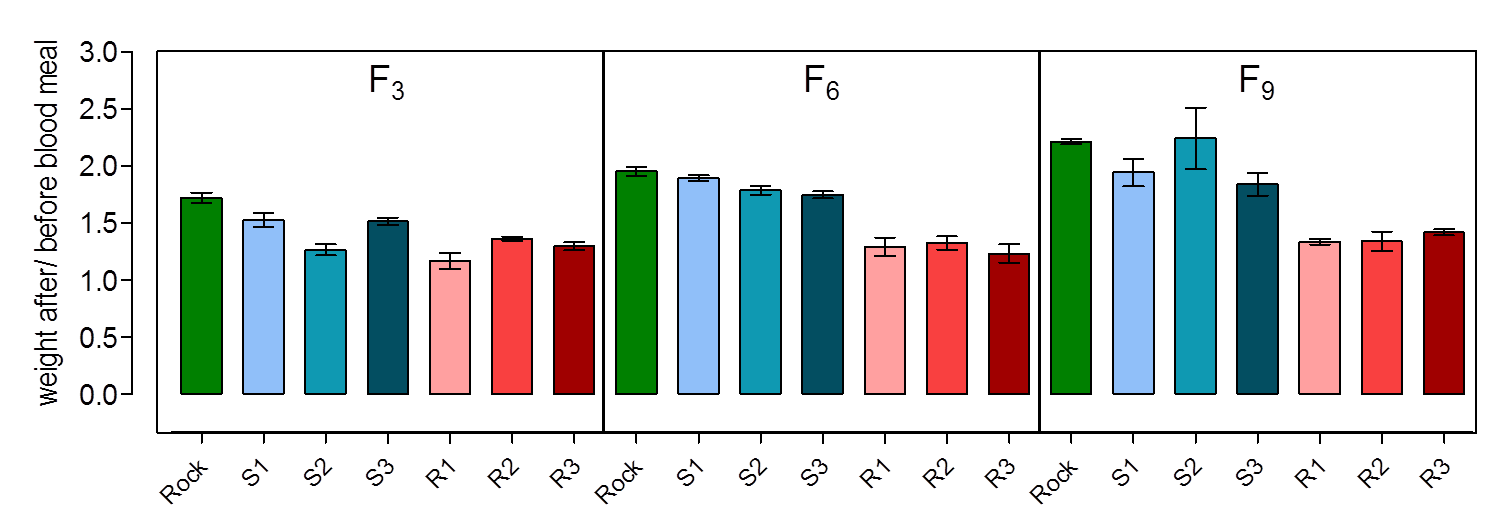

Supplement: Figure S3 — Blood meal ingestion of Ae. aegypti adults resulted from Rock (green) S (blue) and R (red) groups. Mean and standard error are shown. (TIF) [file pone.0031889.s003.tif]
