# Supplementary material for: Effect of Insecticide Resistance on Development, Longevity and Reproduction of Field or Laboratory Selected Aedes aegypti Populations
Source: PLoS One. 2012 Mar 14;7(3):e31889. doi: 10.1371/journal.pone.0031889 (PMC3303777; doi:10.1371/journal.pone.0031889)
Supplement: Table S1 — Student t test comparison between Aedes aegypti pooled R and S groups survival after exposure to deltamethrin under laboratory selection. (DOCX) [file pone.0031889.s004.docx]

**Table S1** – Student *t* test comparison between *Aedes aegypti* pooled R and S groups survival after exposure to deltamethrin under laboratory selection.

| Generation | *t* | df | p |
| --- | --- | --- | --- |
| F3 | 0.980 | 4 | 0.3828 |
| F4 | 1.481 | 4 | 0.2128 |
| F5 | 9.128 | 4 | 0.0008 |
| F8 | 10.320 | 4 | 0.0005 |
| F9 | 8.105 | 4 | 0.0013 |

*t* = t value; df = degrees of freedom; p = probability.
