# Supplementary material for: Effect of Insecticide Resistance on Development, Longevity and Reproduction of Field or Laboratory Selected Aedes aegypti Populations
Source: PLoS One. 2012 Mar 14;7(3):e31889. doi: 10.1371/journal.pone.0031889 (PMC3303777; doi:10.1371/journal.pone.0031889)
Supplement: Table S2 — Aedes aegypti sex ratio of S and R groups under laboratory conditions, throughout generations. (DOCX) [file pone.0031889.s005.docx]

**Table S2** – *Aedes aegypti* sex ratio of S and R groups under laboratory conditions, throughout generations.

|  | **F1** | | | **F3** | | | **F6** | | | **F9** | | |
| --- | --- | --- | --- | --- | --- | --- | --- | --- | --- | --- | --- | --- |
|  | **♀** | **♂** | **p** | **♀** | **♂** | **p** | **♀** | **♂** | **p** | **♀** | **♂** | **p** |
| Rock | 88 | 80 | 0.7434 | 88 | 90 | 1.000 | 108 | 98 | 0.6934 | 89 | 108 | 0.364 |
|  | 97 | 99 | 1.000 | 97 | 93 | 0.9183 | 103 | 102 | 1.000 | 87 | 96 | 0.6758 |
| S1 | 118 | 113 | 0.8524 | 97 | 102 | 0.8411 | 95 | 106 | 0.6187 | 99 | 110 | 0.6247 |
|  | 93 | 114 | 0.3251 | 98 | 122 | 0.2935 | 106 | 98 | 0.7664 | 97 | 102 | 0.8411 |
| S2 | - | - | - | 95 | 111 | 0.4901 | 106 | 105 | 1.000 | 80 | 100 | 0.342 |
|  | - | - | - | 95 | 113 | 0.4324 | 81 | 122 | 0.0464* | 89 | 90 | 1.000 |
| S3 | 100 | 124 | 0.2979 | 89 | 106 | 0.4175 | 123 | 120 | 0.9279 | 97 | 94 | 0.9185 |
|  | 103 | 124 | 0.3475 | 101 | 114 | 0.5627 | 99 | 97 | 1.000 | 100 | 87 | 0.5348 |
| R1 | - | - | - | 105 | 105 | 1.000 | 88 | 108 | 0.3627 | 84 | 94 | 0.6715 |
|  | - | - | - | 75 | 131 | 0.0072** | 90 | 109 | 0.3664 | 80 | 85 | 0.8258 |
| R2 | 74 | 86 | 0.576 | 91 | 117 | 0.2384 | 86 | 114 | 0.1924 | 77 | 96 | 0.3326 |
|  | 140 | 167 | 0.2936 | 113 | 108 | 0.8491 | 95 | 104 | 0.6891 | 100 | 109 | 0.6956 |
| R3 | 111 | 101 | 0.6976 | 116 | 108 | 0.7768 | 86 | 114 | 0.1924 | 89 | 91 | 1.000 |
|  | 87 | 107 | 0.3602 | 98 | 128 | 0.1868 | - | - | - | 77 | 93 | 0.4472 |

Numbers of females and males obtained from S1-S3 and R1-R3 lineages and from Rockefeller strain. For every lineage, each line shows an independent assay. p = probability for Fisher’s exact test; * = p<0.05; ** = p<0.01; - = not done.
