# Supplementary material for: Effect of Insecticide Resistance on Development, Longevity and Reproduction of Field or Laboratory Selected Aedes aegypti Populations
Source: PLoS One. 2012 Mar 14;7(3):e31889. doi: 10.1371/journal.pone.0031889 (PMC3303777; doi:10.1371/journal.pone.0031889)
Supplement: Table S3 — Number of eggs per Aedes aegypti S and R females in the course of selection with deltamethrin. (DOCX) [file pone.0031889.s006.docx]

**Table S3**: Number of eggs per *Aedes aegypti* S and R females in the course of selection with deltamethrin.

| Generation | Median number eggs/female | | | Difference in rank sum for Dunn's Multiple comparison Test | | |
| --- | --- | --- | --- | --- | --- | --- |
|  | Rock | S | R | Rock *Vs* S | Rock *Vs* R | S *Vs* R |
| F1 | 109.5 | 105.5 | 111.0 | 1.370 | -2.247 | -3.617 |
| F3 | 105.5 | 98.0 | 92.5 | -1.066 | 1.402 | 2.467 |
| F6 | 123.0 | 113.5 | 88.5 | 8.582 | 27.02** | 18.43* |
| F9 | 93.5 | 80.0 | 76.0 | 10.07 | 21.22* | 11.14 |
|  | |  |  |  |  |  |

Comparison of number of eggs among Rockefeller and pooled S and R lineages through ANOVA followed by Dunn's Multiple comparison Test. *p<0.05; **p<0.01.
